# Potential role of the skin and gut microbiota in premenarchal vulvar lichen sclerosus: A pilot case-control study

**DOI:** 10.1371/journal.pone.0245243

**Published:** 2021-01-14

**Authors:** Suhana Chattopadhyay, Justin D. Arnold, Leena Malayil, Lauren Hittle, Emmanuel F. Mongodin, Kalyani S. Marathe, Veronica Gomez-Lobo, Amy R. Sapkota

**Affiliations:** 1 Maryland Institute for Applied Environmental Health, University of Maryland School of Public Health, College Park, Maryland, United States of America; 2 George Washington University School of Medicine and Health Sciences, Washington, DC, United States of America; 3 University of Maryland School of Medicine, Institute for Genome Sciences, Baltimore, Maryland, United States of America; 4 Division of Dermatology, Children’s National Health System, Washington, DC, United States of America; 5 Department of Dermatology, University of Cincinnati, Cincinnati, Ohio, United States of America; 6 Pediatric and Adolescent Obstetrics and Gynecology, MedStar Washington Hospital Center/Children’s National Health System, Washington, DC, United States of America; 7 Pediatric and Adolescent Gynecology, National Institute of Child Health and Human Development, Bethesda, Maryland, United States of America; Massachusetts General Hospital, UNITED STATES

## Abstract

The etiology of vulvar lichen sclerosus (LS) remains unclear; however, alterations in cutaneous and gut microbiota may be contributing to the pathogenesis of this inflammatory condition. To explore this hypothesis, we conducted a pilot case-control study, obtaining dermal swab and stool samples from prepubertal girls with vulvar LS (n = 5), girls with nonspecific vulvovaginitis (n = 5), and healthy controls (n = 3). Samples (n = 56) were subjected to total DNA extractions. Resulting DNA was purified, subjected to PCR (targeting the V3V4 region of the 16S rRNA gene), sequenced, and analyzed using QIIME, MetagenomeSeq, and DESeq2 software packages. Our findings showed that there were significant differences in the cutaneous and gut microbiotas of girls with LS compared to controls. On the skin, girls with LS had a statistically significantly higher relative abundance of *Porphyromonas* spp., *Parvimonas* spp., *Peptoniphilus* spp., *Prevotella* spp., *Dialister* spp., *and Peptostreptococcus* spp., but a lower relative abundance of *Cornyebacterium* compared to the control group. In the gut samples, girls with LS had a significantly higher relative abundance of *Dialister* spp., *Clostridiales* spp., *Paraprevotella* spp., *Escherichia coli*, *Bifidobacterium adolescentis*, and *Akkermansia muciniphila*, and a lower relative abundance of *Roseburia faecis* and *Ruminococcus bromii* compared to controls. These results suggest a potential association between cutaneous and gut dysbiosis and pediatric vulvar LS. Future studies involving larger samples sizes are warranted to further evaluate this association.

## Introduction

Lichen sclerosus (LS) is a chronic inflammatory skin condition that presents as painful, pruritic, ivory-white atrophic patches, most commonly within the genital region of premenarchal girls or post-menopausal women [1]. Prolonged untreated disease can lead to scarring, genital disfigurement, and an increased risk of developing squamous cell cancer within the affected areas [2]. Genetic, immunologic, and hormonal factors have been implicated in the pathogenesis of LS, however, the exact etiology of LS remains unclear [3, 4]. Local factors such as the skin microbiota have also been proposed as possible causes to LS, as vulvar skin with LS becomes normal when transplanted to the thigh, and normal skin transplanted to the affected vulva develops LS [5].

Alterations in the skin microbiota have previously been associated with numerous dermatologic conditions, including atopic dermatitis, psoriasis, and hidradenitis suppurativa [6–8]. The skin microbiota likely contributes to the development of dermatologic diseases by disrupting the skin barrier and dysregulating the immune system. For example, *Staphylococcus aureus* is known to express multiple superantigens, including, cytolytic α- and δ-toxins, phenol-soluble modulins, protein A, and several proteases, which likely contribute to the inflammatory state characterizing multiple skin conditions [9]. Additionally, *S*. *aureus* is more prevalent on the skin of infants preceding the development of atopic dermatitis compared to age-matched unaffected infants, suggesting that *S*. *aureus* colonization contributes to the onset of atopic dermatitis and is not simply a result of the disease [10].

Recent studies have also suggested that the gut microbiota may play a role in the pathogenesis of inflammatory skin conditions [11]. The exact mechanisms by which the gut microbiota influences skin homeostasis are still being elucidated, but it appears that the two are linked by the modulatory effect of gut commensals on systemic immunity [12]. Given the inflammatory nature of LS, we hypothesized that alterations in the skin or gut microbiotas may be contributing to the pathogenesis of LS. In order to assess this hypothesis, we performed a pilot case-control study to better characterize the microbiotas of girls with vulvar LS compared to girls with nonspecific vulvovaginitis and healthy controls.

## Materials and methods

### Study design and population

This pilot case-control study compared the cutaneous and gut microbiotas across three groups of premenarchal girls: treatment-naïve girls with a clinical diagnosis of vulvar LS; girls with nonspecific vulvovaginitis (a non-inflammatory condition caused by local irritation); and girls without dermatologic findings (healthy controls). For girls with suspected LS, both a pediatric gynecologist and a pediatric dermatologist participated in the exam and agreed upon the LS diagnosis prior to study enrollment. Subjects were excluded if they had used topical or systemic antibiotics or steroids in the 6 months prior to study enrollment. After obtaining parental written informed consent, a clinical questionnaire was administered and swabs from the perineum and vulva were collected. In addition, stool collection kits were provided for at-home collection. The Children’s National Institutional Review Board approved this study.

### Sample collection

Dry Copan Flocked E-swabs (Copan Diagnostics, Murrieta, CA, USA) were placed between the bilateral labia majora and minora and gently rotated for 5 seconds on each side. A second swab was then placed between the perineum and fourchette outside the hymen and gently rotated for 5 seconds. The swabs were placed in 5 mL of RNALater solution in two separate tubes and stored at -80°C until processed for DNA extraction.

Stool collection kits were provided to parents with instructions on how to collect stool at home. Briefly, parents were instructed to have children deposit the stool (without urine) directly into the Fisherbrand Commode Specimen Collection System (Fisher Scientific, Waltham, MA, USA), before scooping the stool into a Norgen Biotek Stool Collection Tube (Norgen Biotek Corp, Thorold, ON, CA) containing a nucleic acid preservation buffer, enabling the sample to be shipped at room temperature. Another portion of stool was then scooped into an empty VWR specimen container (VWR, Radnor, PA, USA) and samples were subsequently shipped (at room/ambient temperature) to study investigators where they were stored at -80°C.

### DNA extraction and purification

All samples were thawed at 4°C before they were opened under sterile conditions. Tubes containing RNALater and swabs were vortexed for 10 sec, and then the tip of the swabs were cut and transferred to lysing matrix tubes. The remaining 5 mL of RNALater solution was transferred to another lysing matrix tube. After vortexing, 200 mL of the liquid stool sample from the Norgen Biotek Stool Collection Tubes was pipetted out into lysing matrix tubes. Similarly, 0.2 g of each solid stool sample was weighed out and placed into another lysing matrix tube. Total genomic DNA was extracted from each sample using a protocol published previously [13]. DNA was further purified from the lysate using the QIAmp DSP DNA mini kit 50, v2 (Qiagen, Hilden, DE) per the manufacturer’s protocol.

### 16S ribosomal RNA gene PCR amplification and sequencing

The V3V4 region of the 16S ribosomal RNA gene was amplified and sequenced using the 319F (ACTCCTACGGGAGGCAGCAG) and 806R (GGACTACHVGGGTWTCTAAT) universal primers. Primers included barcodes for each sample, as well as a linker sequence required for Illumina HiSeq 2500 300-bp paired-ends sequencing and a 12-bp heterogeneity spacer index sequence. Amplification of sample DNA and negative controls was completed using thermocycler parameters described previously [13]. Resulting amplicons were sequenced on the Illumina HiSeq 2500 (Illumina, San Diego, CA, USA) using protocols previously published [14].

### Sequence quality filtering and analysis

16S ribosomal RNA gene reads were initially screened for low quality and short length, then assembled using PANDAseq, demultiplexed and chimera trimmed using UCHIME [15, 16]. 16S ribosomal RNA gene sequences were then processed using QIIME v1.9 where de-novo operational taxonomic unit (OTU) clustering was performed using VSEARCH, and taxonomic assignments were performed using the GreenGenes v.13.8 reference database [17]. Downstream analyses were then performed within RStudio (v. 0.99.473) where the phyloseq package (v. 1.19.1) was used for alpha- and beta-diversity analyses, and the ggplot2 package (v. 2.2.1) was used for visualization [18]. Alpha diversity analysis was performed on rarefied reads. Data normalization was performed using cumulative sum scaling with the MetagenomeSeq (v. 1.16.0) package [19]. Significance in beta-diversity comparisons was tested using the vegan package [18, 20]. Statistically significant differences (*p* value cutoff of 0.05) in bacterial OTU relative abundance across subject groups by sample type were calculated using DESeq2 package (alpha = 0.05) [21] on OTUs >0.1% abundance.

## Results

### Study population characteristics

A total of 13 subjects were included in the pilot study: 5 patients with LS, 5 patients with nonspecific vulvovaginitis; and 3 healthy controls (Table 1). Four of the 5 LS patients had "severe" or "very severe" disease as assessed by study clinicians, and all 5 LS patients had ≥50% involvement of at least 1 of the 5 sites (clitoral hood, labia minora, labia majora, perineum, or perianal region) (Table 1). No patients had genital mutilation. The mean age was similar across the three groups, but there was some variability in race and ethnicity. In regards to breastfeeding, 80% of girls with LS had been exclusively breastfed for more than two months compared to no subjects with nonspecific vulvovaginitis and 33% of healthy controls (Table 1).

**Table 1:** Characteristics of study participants.

Characteristic	Lichen Sclerosus (n = 5)	Nonspecific vulvovaginitis* (n = 5)	Healthy Controls (n = 3)
Age, mean (SD), years	6 (2.2)	6.4 (2.3)	6.3 (0.6)
White race/ethnicity, n (%)	2 (40)	3 (60)	3 (100)
Black race/ethnicity, n (%)	3 (60)	2 (40)	0
Hispanic race/ethnicity, n (%)	0	3 (60)	1 (33)
Birth method, n (%)			
Vaginal with epidural	3 (60)	0	2 (67)
Vaginal without epidural	1 (20)	1 (25)	1 (33)
Cesarean section	1 (20)	3 (75)	0
≥50% involvement, n (%)			
Clitoral hood	3 (60)	NA	NA
Labia majora	4 (80)	NA	NA
Labia minora	3 (60)	NA	NA
Perineum	5 (100)	NA	NA
Perianal	3 (60)	NA	NA
Disease duration, mean (SD), months	14.4 (12.4)	NA	NA
Genital adhesions/loss architecture	0	NA	NA
Family history LS	0	0	0
Family history autoimmune disorder	1 (20)	1 (25)	0
Exclusively breastfed ≥ 2 months, n (%)	4 (80)	0	1 (33)
Store-bought diapers, n (%)	5 (100)	4 (100)	3 (100)
Baths at least weekly, n (%)	4 (80)	2 (50)	2 (67)
Symptoms, n (%)			
Constipation	3 (60)	3 (75)	0
Dysuria	1 (20)	2 (50)	0
Pruritus	3 (60)	1 (25)	0
Incontinence	1 (20)	1 (25)	0
Painful defecation	1(20)	2 (50)	0
Abdominal pain	1(20)	2 (50)	1 (33)
Nausea	0 (0)	1 (25)	0

*One patient did not answer clinical questionnaire.

### Sequencing dataset

A total of 56 samples from 13 subjects were sequenced: perineal swabs (n = 12) and swab solutions (n = 12), labia majora and minora swabs (n = 12) and swab solutions (n = 10), as well as liquid (n = 5) and solid stool samples (n = 5). A total of 2,639,624 sequences were generated with an average of 47,136 sequences (standard deviation 31,096) per sample. The maximum number of reads per sample was 104,638 and the minimum number of reads was 531. To ensure appropriate sequence coverage for each sample, a Good’s coverage cutoff was set at 0.90 (S1 Fig). After quality filtering and eliminating samples with reads falling below the 0.90 Good’s coverage cutoff, a total of 2,639,093 reads were obtained from 55 samples for downstream analysis, with a maximum of 104,638 and minimum of 798 reads per sample (S1 Table).

The average number of reads from the skin samples of LS patients was 49,866.87 with an average of 643.81 OTUs and an average Good’s coverage of 0.99. The average number of reads from the skin samples of patients with nonspecific vulvovaginitits was 43,936.95 with an average of 486.22 OTUs and an average Good’s coverage of 0.99. The average number of reads from the skin samples of controls was 39,715.14 with an average of 525.71 OTUs and an average Good’s coverage of 0.99. There were no significant differences between diversity indices on rarefied reads (both alpha and beta diversity) between skin (labial and perineum samples); therefore, these samples were combined (S2 Fig). Diversity indices also did not indicate statistically significant differences between swab and swab solutions for skin samples, or between solid and liquid stools samples. Hence, we merged each participant’s swab and swab solutions from skin samples, and solid and liquid stool samples, resulting in a total of 45 skin samples and 10 stool samples. Overall, sequences were clustered into 5,466 operational taxonomic units (OTUs). Of these OTUs, 98% were assigned to a phylum level, 28% to a genus level and 4.5% to a species level.

### Microbiota differences between LS and healthy subjects

**Fig 1:**
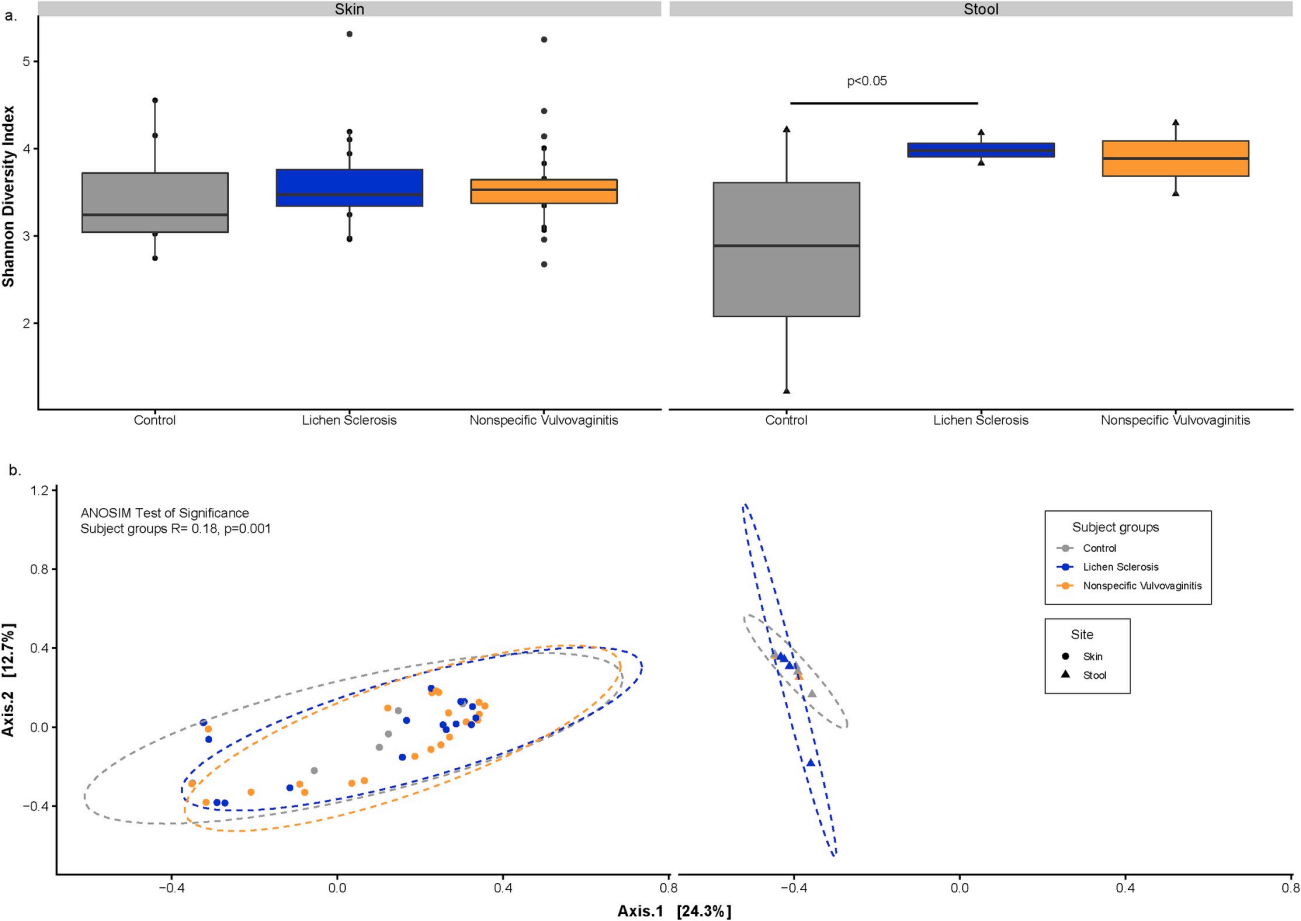
Bacterial diversity between subject groups and sample types. [a] Boxplots showing alpha diversity (Shannon Index) across all sample types; and [b] Principal coordinates analysis plots of Bray-Curtis computed distances between sample types. Ellipses represent 95% confidence intervals.

Alpha diversity using the Shannon diversity metric was measured between subject groups by sample type (Fig 1A). Significant differences were observed in Shannon diversity between the gut microbiota of patients with LS compared to healthy controls (*P*<0.05). Significant differences were not observed in skin samples from LS patients compared to the control samples. Beta diversity analyses was computed using principal coordinates analysis (PCoA) of Bray-Curtis dissimilarity using CSS-normalized (non-rarefied) dataset. This analysis indicated that the subject groups (LS, nonspecific vulvovaginitits, and controls) explained 18.08% of the variability (ANOSIM *P*<0.001), and sample type (skin and stool) explained 43.91% (ANOSIM *P*<0.001) of the variability observed in the bacterial communities (Fig 1B).

**Fig 2:**
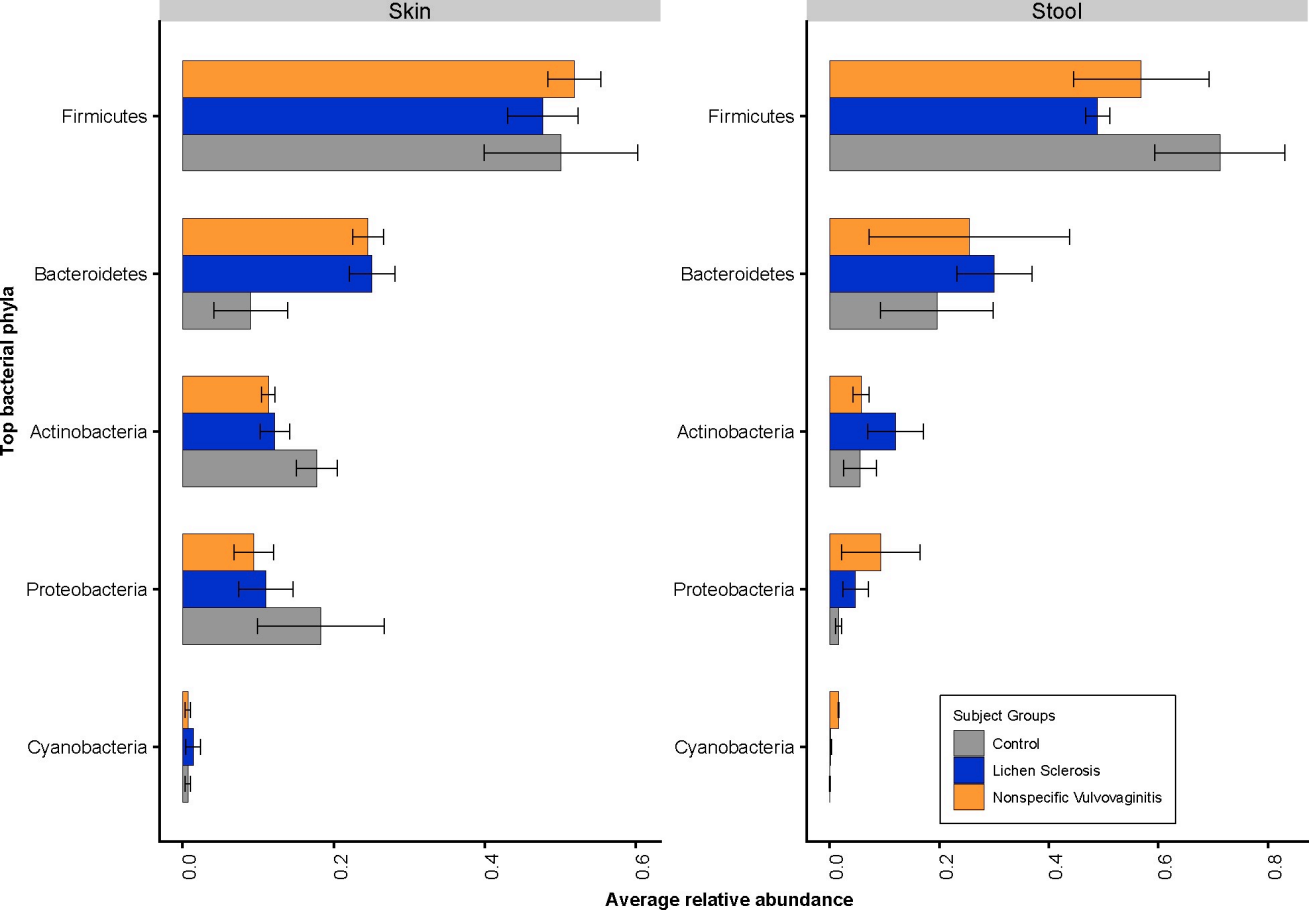
Relative abundance of top five bacterial phyla across skin and stool samples.

In terms of bacterial taxa composition, the skin and gut of all subjects were dominated at the phylum level by *Firmicutes*, followed by *Bacteroidetes*, *Actinobacteria*, and *Proteobacteria* (Fig 2). Comparing within the skin samples, patients with LS had a lower relative abundance of *Firmicutes*, *Actinobacteria*, *and Proteobacteria*, and a higher relative abundance of *Bacteroidetes* compared to healthy controls. Comparing within stool samples, patients with LS had a lower relative abundance of *Firmicutes*, and a higher relative abundance of *Bacteroidetes*, *Actinobacteria* and *Proteobacteria* compared to healthy controls.

In total, 1,545 OTUs were classified to the genus level, and among those, 247 were identified to the species level. There were significantly different bacterial genera identified between stool and skin samples (S3 Fig), and between subject groups (S4 Fig). Additionally, there were bacterial genera that were shared/overlapped between subject groups and sample types, forming core microbiomes for those sets of samples. Specifically, there were 42 OTUs that were shared between subject groups, and 34 OTUs shared between sample types (S2 and S3 Tables, respectively).

**Fig 3:**
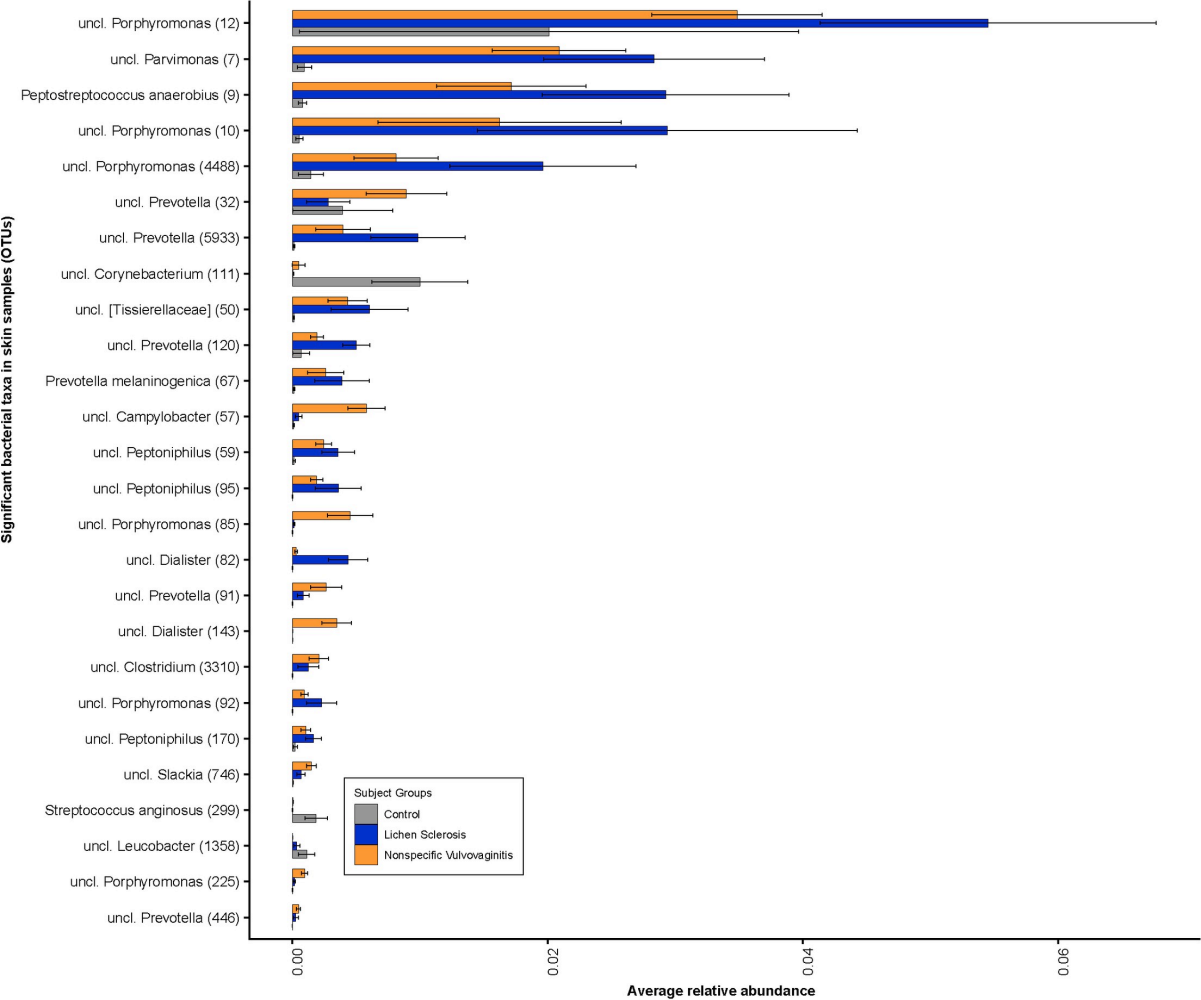
Bacterial community composition in skin samples. Relative abundance plots of top bacterial species computed using differentially abundant (*P*<0.05) OTUs comparing between the subject groups. Number in parentheses denotes OTU ID.

At the genus level, the anogenital skin of all subject groups was dominated by *Porphyromonas* spp., *Prevotella* spp., *Corynebacterium* spp., and *Peptoniphilus* spp. (Fig 3). Comparing between subject groups, the skin microbiota of girls with LS had a higher relative abundance of the bacterial genera *Porphyromonas* spp. (OTU#12, OTU#10, OTU#4488, OTU#92), *Parvimonas* spp. (OTU#7), *Peptoniphilus* spp. (OTU#59, OTU#95 and OTU#170), *Prevotella* spp. (OTU#5933, OTU#120), *Dialister* spp. (OTU#82) and *Peptostreptococcus* spp. (OTU#9), but a lower relative abundance of *Corynebacterium* spp. (OTU#111) compared to control subjects. *Campylobacter* spp. (OTU#57) and *Dialister* spp. (OTU#143) was highest in the skin samples from nonspecific vulvovaginitis group. At the species level, the skin samples of girls with LS and nonspecific vulvovaginitis had a significantly lower relative abundance of *Streptococcus anginosus* (OTU#299), but a significantly higher abundance of *Peptostreptococcus anaerobius* (OTU#9) and *Prevotella melaninogenica* (OTU#67) when compared to that of the controls. Comparing across all three subject groups, among 26 significant bacterial genera, only three taxa (*Corynebacterium* (OTU#111), *Streptococcus anginosus* (OTU#299), and *Leucobacter* OTU#1358) were at a higher relative abundance in the control group. Overall, the lowest abundance of bacteria was found in the skin samples from the control group.

**Fig 4:**
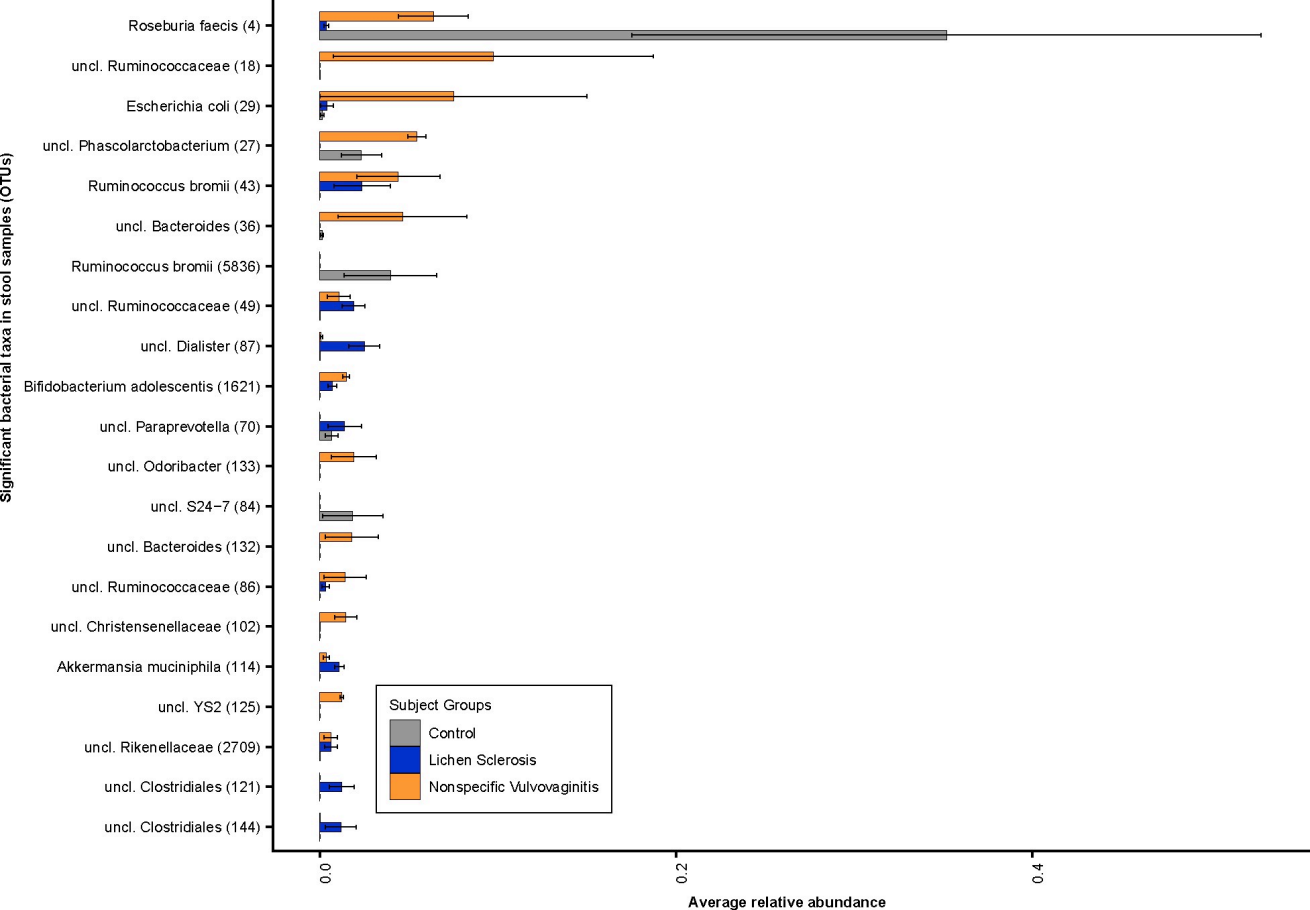
Bacterial community composition in stool samples. Relative abundance plots of top bacterial species computed using differentially abundant (*P*<0.05) OTUs comparing between the subject groups. Number in parentheses denotes OTU ID.

Comparing stool samples across all subject groups, girls with LS had a higher relative abundance of the bacterial genera *Dialister* spp. (OTU#87), *Clostridiales* spp. (OTU#121 and OTU#144), *Paraprevotella* spp. (OTU#70) and *Escherichia coli* (OTU#29) but a lower relative abundance of *Phascolarctobacterium* spp. (OTU#27) compared to healthy controls (Fig 4). Furthermore, at the species level, girls with LS and nonspecific vulvovaginitis had a significantly lower relative abundance of *Roseburia faecis* (OTU#4) and *Ruminococcus bromii* (OTU#5836) and a higher relative abundance of *Bifidobacterium adolescentis* (OTU#1621) and *Akkermansia muciniphila* (OTU#114) compared to healthy individuals. Girls with nonspecific vulvovaginitis had a significantly higher relative abundance of *Escherichia coli* (OTU#29) and *Bifidobacterium adolescentis* (OTU#1621) in the gut compared to the healthy controls.

## Discussion

We demonstrated that girls with LS have a higher relative abundance of *Dialister* spp. in the gut, which is consistent with findings from other inflammatory diseases [22]. *Dialister* spp. abundance has been suggested to be a potential microbial marker of disease activity in ankylosing spondyloarthritis, as an increasing abundance of *Dialister* spp. in the stool of patients with spondyloarthritis correlated with worsening Ankylosing Spondylitis Disease Activity Scores [22].

Conversely, girls with LS had a lower relative abundance of *Roseburia faecis* in the gut compared to healthy controls and girls with nonspecific vulvovaginitis. *Roseburia* spp. are commensal bacteria that produce short-chain fatty acids, most notably butyrate, which has been shown to improve colonic motility, immunity maintenance, and provide anti-inflammatory effects [23]. For example, butyrate is the main energy source for the colonic epithelium and inhibits expression of proinflammatory cytokines in the colonic mucosa by preventing NF-κB activation [24]. Clinically, multiple studies have noted a decrease in ileal and colonic *Roseburia* spp. in patients with Crohn’s disease and ulcerative colitis, respectively [25–27]. Patients with inflammatory bowel disease (IBD) have also been observed to have lower concentrations of stool butyrate, providing further evidence to suggest a possible causative role of gut dysbiosis in inflammatory conditions such as IBD and possibly pediatric vulvar LS [28].

The patterns of cutaneous dysbiosis observed in this study have also been implicated in other inflammatory skin conditions. Consistent with the microbiota of psoriatic lesions, the bacterial genera *Prevotella* spp. and *Peptostrepococcus* spp. were more abundant on the anogenital skin of girls with LS compared to healthy individuals [29, 30]. Girls with LS also had a higher abundance of *Porphyromonas* spp. and *Parvimonas* spp. on the skin*—*bacterial genera repeatedly shown to be in higher abundance in hidradenitis suppurativa lesions [7, 31].

Interestingly, the pattern of cutaneous dysbiosis observed here—predominated by the anaerobic genera *Prevotella* spp., *Porphyromonas* spp., and *Parvimonas* spp.*—*has previously been implicated in the pathogenesis of chronic periodontitis, as these bacteria are also found in the oropharynx in addition to the vagina [32]. In chronic periodontitis, these anaerobic organisms use sophisticated strategies to evade immune-regulated killing and to induce a favorable inflammatory state. For example, *Porphyromonas* spp. and *Prevotella* spp. evade the immune system by secreting the bacterial proteases gingipain and interpain A, respectively [33, 34]. These bacterial proteases cleave complement, thereby, inhibiting opsonization and phagocytosis, and allowing bacterial overgrowth which increases inflammation. These bacterial genera subsequently utilize the local inflammation to acquire nutrients from tissue breakdown products, such as degraded collagen peptides and heme containing compounds [35]. Similar inflammatory mechanisms may be contributing to the pathogenesis of vulvar LS given the similarities in dysbiosis observed between the skin of girls with LS and chronic periodontitis.

The etiology of the observed dysbiosis remains unclear. Girls with LS in this study were more likely to be breast fed, which is typically associated with a more beneficial gut microbiota [36]. Exclusively breastfed infants, however, are at risk for vitamin D deficiency even into early childhood [37]. Vitamin D deficiency has been shown to not only negatively affect the gut microbiota, but also affect the onset and activity of IBD [38–40]. These results raise the possibility that vitamin D deficiency caused by breast feeding in infancy, may be affecting the gut and skin microbiota, thereby, increasing one’s risk for developing LS. On the other hand, a case report of a patient with cutaneous LS demonstrated that treatment with an oral daily dose of 0.5 mcg calcitriol (1–25 dihydroxyvitamin D3) over 6 months resulted in dramatic improvements, with skin extensibility increasing and lesions improving [41]. Hence, further research is necessary to assess: 1) if the patterns of breastfeeding observed here are present in larger cohorts; 2) if children who develop LS have lower vitamin D levels than healthy controls; and 3) if oral treatment with vitamin D3 can help alleviate LS symptoms in additional individuals.

This study has multiple limitations. First, this study is limited by its small sample size. Study personal found it difficult to recruit pediatric patients and families amenable to collecting stool specimens—incentives are likely required in future trials to increase participation. Second, girls with LS lacked histopathologic confirmation of the LS diagnosis. However, the diagnosis of LS in this study was made jointly upon physical examination by both a pediatric dermatologist and a pediatric gynecologist whom specialize in pediatric anogenital disorders. Third, many of the observed microorganisms in this study could not be assigned to the species level. To increase species and strain level resolution in future studies, the V1-V3 regions of the bacterial 16S ribosomal RNA gene could be sequenced or metagenomic shotgun sequencing could be performed [42]. Finally, this study was not designed to characterize yeasts, viruses, and other microeukaryotes, such as *Demodex* spp., within cutaneous or gut microbiotas. When reference genomes for these microorganisms become available, it is important to characterize their relative abundance and diversity in clinical samples as they may also be contributing to the development of skin disease.

In conclusion, we demonstrated that there are significant alterations in the cutaneous and gut microbiotas of girls with LS. These results—which are consistent with findings regarding other inflammatory diseases—suggest that skin and gut dysbiosis may be involved in the pathogenesis of pediatric vulvar LS. Further research, however, is necessary to assess whether these microbiota changes are causative or simply a result of the underlying disease. Additionally, further studies are needed to evaluate the potential benefits of alternative therapeutics, such as prebiotics, probiotics, and vitamin D supplementation, in the management of LS.

## Supporting information

S1 Fig Scatterplot of number of sequences and Good’s coverage across all skin and stool samples. (DOCX)

S2 Fig Bacterial diversity between labia majora and minora samples and perineum samples. (DOCX)

S3 Fig Relative abundances of bacterial OTUs that were statistically significantly different (p<0.05) between skin and stool samples. A positive log2-fold change value denotes an OTU that is significantly higher in skin samples, while a negative log2-fold change indicates an OTU that is significantly higher in stool samples. The grey line and arrows highlight the conversion in log2-fold change from negative to positive values.
(DOCX)

S4 Fig Relative abundances of bacterial OTUs that were statistically significantly different (p<0.05) between subject groups within A) stool and B) skin samples. Left panel: A positive log2-fold change value denotes an OTU that is significantly higher in LS patients, while a negative log2-fold change indicates an OTU that is significantly higher in non-specific vulvovaginitis group. Central panel: A positive log2-fold change value denotes an OTU that is significantly higher in healthy controls, while a negative log2-fold change indicates an OTU that is significantly higher in LS patients. Right panel: A positive log2-fold change value denotes an OTU that is significantly higher in healthy controls, while a negative log2-fold change indicates an OTU that is significantly higher in non-specific vulvovaginitis. The grey line highlights the conversion in log2-fold change from negative to positive values.
(DOCX)

S1 Table Bacterial OTU abundance in all samples. (XLSX)

S2 Table OTUs (core microbiome) that were shared between subject groups (Lichen sclerosus, non-specific vulvovaginitis, healthy controls). (XLSX)

S3 Table OTUs (core microbiome) that were shared between sample types (stool and skin). (XLSX)
