# Supplementary material for: Potential role of the skin and gut microbiota in premenarchal vulvar lichen sclerosus: A pilot case-control study
Source: PLoS One. 2021 Jan 14;16(1):e0245243. doi: 10.1371/journal.pone.0245243 (PMC7808574; doi:10.1371/journal.pone.0245243)
Supplement: S1 Fig — (DOCX) [file pone.0245243.s001.docx]

**S1 Fig.** Scatterplot of number of sequences and Good’s Coverage across all skin and stool samples.
