# Supplementary material for: Potential role of the skin and gut microbiota in premenarchal vulvar lichen sclerosus: A pilot case-control study
Source: PLoS One. 2021 Jan 14;16(1):e0245243. doi: 10.1371/journal.pone.0245243 (PMC7808574; doi:10.1371/journal.pone.0245243)
Supplement: S2 Fig — (DOCX) [file pone.0245243.s002.docx]

**S2 Fig.** Bacterial diversity between labia majora and minora samples and perineum samples.
