# Supplementary material for: Potential role of the skin and gut microbiota in premenarchal vulvar lichen sclerosus: A pilot case-control study
Source: PLoS One. 2021 Jan 14;16(1):e0245243. doi: 10.1371/journal.pone.0245243 (PMC7808574; doi:10.1371/journal.pone.0245243)

**S3 Fig.** Relative abundances of bacterial OTUs that were statistically significantly different (p<0.05) between skin and stool samples. A positive log2-fold change value denotes an OTU that is significantly higher in skin samples, while a negative log2-fold change indicates an OTU that is significantly higher in stool samples. The grey line and arrows highlight the conversion in log2-fold change from negative to positive values.


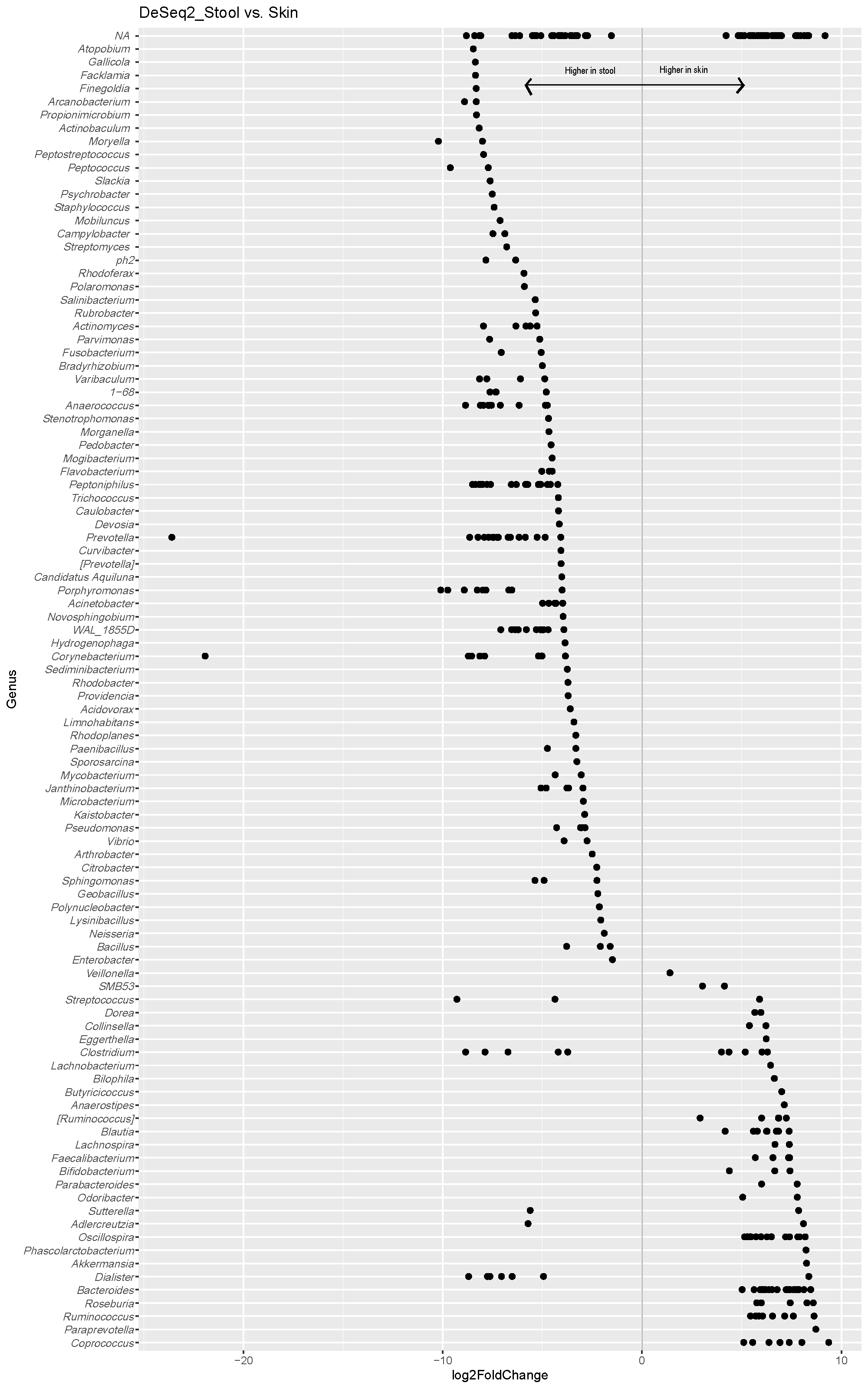

Supplement: S3 Fig — A positive log2-fold change value denotes an OTU that is significantly higher in skin samples, while a negative log2-fold change indicates an OTU that is significantly higher in stool samples. The grey line and arrows highlight the conversion in log2-fold change from negative to positive values. (DOCX) [file pone.0245243.s003.docx]
