# Supplementary material for: Potential role of the skin and gut microbiota in premenarchal vulvar lichen sclerosus: A pilot case-control study
Source: PLoS One. 2021 Jan 14;16(1):e0245243. doi: 10.1371/journal.pone.0245243 (PMC7808574; doi:10.1371/journal.pone.0245243)

**S4 Fig.** Relative abundances of bacterial OTUs that were statistically significantly different

(p<0.05) between subject groups within A) stool and B) skin samples. Left panel: A positive

log2-fold change value denotes an OTU that is significantly higher in LS patients, while a

negative log2-fold change indicates an OTU that is significantly higher in non-specific

vulvovaginitis group. Central panel: A positive log2-fold change value denotes an OTU that is

significantly higher in healthy controls, while a negative log2-fold change indicates an OTU that

is significantly higher in LS patients. Right panel: A positive log2-fold change value denotes an

OTU that is significantly higher in healthy controls, while a negative log2-fold change indicates

an OTU that is significantly higher in non-specific vulvovaginitis. The grey line highlights the

conversion in log2-fold change from negative to positive values.


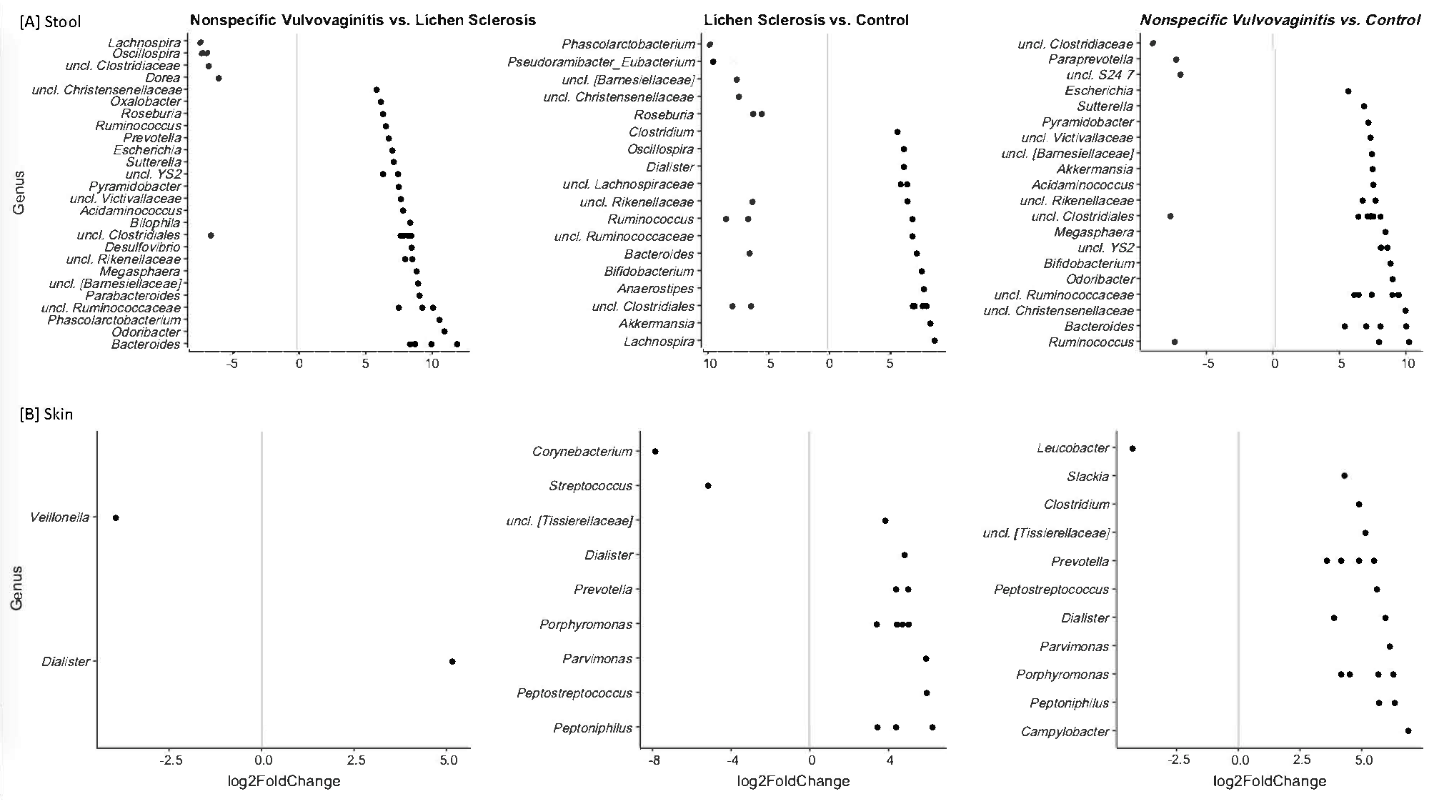

Supplement: S4 Fig — Relative abundances of bacterial OTUs that were statistically significantly different (p<0.05) between subject groups within A) stool and B) skin samples. Left panel: A positive log2-fold change value denotes an OTU that is significantly higher in LS patients, while a negative log2-fold change indicates an OTU that is significantly higher in non-specific vulvovaginitis group. Central panel: A positive log2-fold change value denotes an OTU that is significantly higher in healthy controls, while a negative log2-fold change indicates an OTU that is significantly higher in LS patients. Right panel: A positive log2-fold change value denotes an OTU that is significantly higher in healthy controls, while a negative log2-fold change indicates an OTU that is significantly higher in non-specific vulvovaginitis. The grey line highlights the conversion in log2-fold change from negative to positive values. (DOCX) [file pone.0245243.s004.docx]
